# Biochemical Reconstitution of Hemorrhagic-Fever Arenavirus Envelope Glycoprotein-Mediated Membrane Fusion

**DOI:** 10.1371/journal.pone.0051114

**Published:** 2012-11-30

**Authors:** Celestine J. Thomas, Sundaresh Shankar, Hedi E. Casquilho-Gray, Joanne York, Stephen R. Sprang, Jack H. Nunberg

**Affiliations:** 1 Center for Biomolecular Structure and Dynamics, The University of Montana, Missoula, Montana, United States of America; 2 Division of Biological Sciences, The University of Montana, Missoula, Montana, United States of America; 3 Montana Biotechnology Center, The University of Montana, Missoula, Montana, United States of America; University of Missouri, United States of America

## Abstract

The membrane-anchored proteins of enveloped viruses form labile spikes on the virion surface, primed to undergo large-scale conformational changes culminating in virus-cell membrane fusion and viral entry. The prefusion form of these envelope glycoproteins thus represents an important molecular target for antiviral intervention. A critical roadblock to this endeavor has been our inability to produce the prefusion envelope glycoprotein trimer for biochemical and structural analysis. Through our studies of the GPC envelope glycoprotein of the hemorrhagic fever arenaviruses, we have shown that GPC is unique among class I viral fusion proteins in that the mature complex retains a stable signal peptide (SSP) in addition to the conventional receptor-binding and transmembrane fusion subunits. In this report we show that the recombinant GPC precursor can be produced as a discrete native-like trimer and that its proteolytic cleavage generates the mature glycoprotein. Proteoliposomes containing the cleaved GPC mediate pH-dependent membrane fusion, a characteristic feature of arenavirus entry. This reaction is inhibited by arenavirus-specific monoclonal antibodies and small-molecule fusion inhibitors. The *in vitro* reconstitution of GPC-mediated membrane-fusion activity offers unprecedented opportunities for biochemical and structural studies of arenavirus entry and its inhibition. To our knowledge, this report is the first to demonstrate functional reconstitution of membrane fusion by a viral envelope glycoprotein.

## Introduction

Entry of enveloped viruses into their host cells requires fusion of the viral and cellular membranes, a process that is mediated by the viral envelope glycoprotein. Class I viral fusion proteins, including those of influenza and human immunodeficiency virus type 1 (HIV-1), are synthesized as inactive precursor glycoproteins that assemble as trimers and are subsequently primed by proteolytic cleavage to generate the mature fusogenic spikes. The membrane-anchored spike is thought to exist in a kinetically trapped metastable state that can be triggered, by engagement with cell-surface receptor or exposure to acidic pH in the endosome, to undergo a series of structural transitions leading to a thermodynamically favored postfusion state and concomitant virus-cell membrane fusion (reviewed in references [1], [2]). Intervention strategies that prevent membrane fusion and virus entry thus provide a sound basis for vaccine and drug development. A detailed mechanistic understanding of viral membrane fusion and its inhibition has been hindered by the inherent instability of the prefusion envelope glycoprotein trimer. Solubilization from its membrane-anchored environment invariably causes disassembly and/or refolding to the postfusion conformation. X-ray crystallographic analyses of the most extensively characterized class I envelope glycoproteins – influenza virus hemagglutinin (HA), HIV-1 envelope glycoprotein (Env) and parainfluenza virus 5 F (PIV5 F) - are based on soluble ectodomain fragments. These studies necessarily exclude information regarding the important role of membrane anchorage in envelope glycoprotein assembly, maintenance of the prefusion state and activation of fusogenic conformational changes. The failure of current HIV-1 vaccines to elicit broadly neutralizing antibodies is largely attributed to our inability to produce the trimeric native Env immunogen in a prefusion conformation [3].

Arenaviruses are responsible for severe hemorrhagic fevers worldwide, and Junín (JUNV) and Lassa (LASV) viruses are recognized to pose significant threats to public health and biodefense [4]–[8]. Arenavirus entry into the host cell is mediated by the viral envelope glycoprotein GPC, a member of the class I viral fusion proteins. The GPC precursor trimerizes and is proteolytically cleaved by the cellular site-1-protease/subtilisin-like kexin isozyme-1 (S1P/SKI-1) [9]–[11] in the Golgi to generate the receptor-binding (G1) and transmembrane fusion (G2) subunits. Upon engaging a cell-surface receptor – transferrin receptor 1 (TfR1) for JUNV [12] or alpha-dystroglycan for LASV [13] - the virion is endocytosed and GPC-mediated fusion is triggered by acidic pH in the maturing endosome [14]. The ensuing conformational changes are driven by formation of the stable postfusion trimer-of-hairpins in G2 [15]–[17]. Unlike other class I fusion proteins, the mature GPC retains its signal peptide as an essential subunit [18], [19]. The unusually long (58 amino-acid residues) stable signal peptide (SSP) traverses the membrane twice [20] and binds the cytoplasmic domain of G2 via an intersubunit zinc finger [21], [22]. Evidence suggests that SSP interacts with the ectodomain of G2 to maintain the prefusion GPC complex at neutral pH and facilitate its fusogenic response to acidic pH [23]. Importantly, small-molecule fusion inhibitors [24]–[26] target the pH-sensitive SSP-G2 interface to prevent fusion of the viral and endosomal membranes, and thereby viral entry [23].

Our previous studies showed that the JUNV GPC precursor purified from insect cells exists as a stable trimer and efficiently binds the TfR1 receptor and arenavirus-specific small-molecule fusion inhibitors [27]. The unusual structural integrity of the precursor likely reflects its unique tripartite organization, and suggested the feasibility of generating the mature GPC complex for biochemical analysis. To this end, we have produced the prefusion GPC trimer through *in vitro* proteolytic cleavage, and demonstrated that proteoliposomes containing this complex are able to mediate pH-dependent membrane fusion that is specifically inhibited by small-molecule fusion inhibitors. Biochemical reconstitution of the fusogenic activity of GPC provides a platform for understanding pH-induced membrane fusion and its inhibition.

## Materials and Methods

### Monoclonal Antibodies (MAbs) and Small-molecule Fusion Inhibitors

MAbs directed to JUNV G1 (BF11, BF09, BE08 and AG02) and N (BG12) were obtained from the CDC [28] through the NIAID Biodefense and Emerging Infectious Diseases Research Resources Repository. MAb F100G5 recognizes the fusion peptide of JUNV G2 [16] and was provided by the Public Health Agency of Canada. Plasma-derived human soluble TfR (sTfR) was obtained from American Research Products, and the M2 anti-FLAG MAb from Sigma.

The small-molecule fusion inhibitors discovered by SIGA Technologies - ST-193 [25], ST-161 [26], ST-761 [27], ST-294 [24] and its dansyl analog ST-375 [27] -were obtained from the company. Compounds discovered at The Scripps Research Institute (TSRI) - 17C8 and 8C1 [29] - were provided by Stefan Kunz, Andrew M. Lee and Michael B. A. Oldstone. ST-294 and ST-761 are specific to New World (NW) arenaviruses and ST-161 and TSRI 8C1 are specific to the Old World (OW) LASV. ST-193 and TSRI 17C8 are broadly active against NW and OW viruses.

### Expression and Purification of rGPC^fur^

Recombinant baculoviruses were constructed as previously described [27] using a pFastBac Dual expression vector (Life Technologies) and separate SSP and G1G2 precursor open-reading frames from GPC of the pathogenic MC2 strain of JUNV [19]. These two polypeptides associate in *trans* to reconstitute the native GPC complex [18], [19], [27]. The signal peptide of human CD4 was fused to the N-terminus of the G1G2 precursor, to which a C-terminal extension bearing a TEV-protease site and FLAG-tag sequence was added [27]. The furin recognition motif was introduced using QuikChange mutagenesis (Stratagene). Baculovirus expressing rGPC^fur^ was used to infect *Trichoplusia ni* High-Five™ cells and washed cell membranes were solubilized in 1.5% dodecyl beta-D-maltoside (DDM) as previously described [27]. rGPC^fur^ was bound to immobilized M2 anti-FLAG MAb beads (Sigma), and eluted in buffer containing 0.1% DDM and 5 µM 3xFLAG peptide (Sigma). This material was dialyzed to remove the 3xFLAG peptide and subjected to size-exclusion chromatography using a Superdex-200/G-75 tandem column (GE Healthcare) in buffer containing 0.05% DDM. All buffers contained 100 µM Zn^++^ to prevent dissociation from the intersubunit zinc-binding domain of GPC. Proteins were analyzed by sodium dodecylsulfate-polyacrylamide gel electrophoresis (SDS-PAGE) using NuPAGE 4–12% bis-Tris gels (Life Technologies). For mass spectrometry, proteins were eluted from gels in 0.15 M NaOH and subsequently neutralized and deglycosylated using PNGase F (New England Biolabs). Molecular weights were determined using a MALDI (Voyager DE) mass spectrometer.

### Soluble Human Furin (sFurin)

A plasmid for baculovirus expression of the recombinant soluble prodomain of human furin [30] was kindly provided by Dr. Alex Strongin (Burnham Institute for Medical Research) and the open-reading frame, including the baculovirus gp67 signal peptide and C-terminal hexa-histidine tag, was transferred to pFastBac1 (Life Technologies) for generating baculovirus. The soluble furin enzyme was purified from the supernatant of infected *Spodoptera frugiperda* (Sf9) cells using Ni^++^-affinity chromatography as described [30]. The purified sFurin (∼33,000 units/mg [31]) contained no detectable nonspecific protease activity. Cleavage of rGPC^fur^ in solution (0.6 mg/ml) was performed using a 3∶1 molar ratio of rGPC^fur^:sFurin at 20°C for 4 hr in buffer containing 50 mM Tris (pH 7.2), 150 mM NaCl, 5 mM MgCl_2_, 1 mM CaCl_2_, 100 µM ZnSO_4_ and 0.05% DDM. Zn^++^ was present to stabilize the intersubunit zinc-binding domain in GPC and did not appear to reduce the extent of furin cleavage [32]. In preliminary studies, soluble human furin was purchased from New England Biolabs.

### Surface Plasmon Resonance (SPR) Studies

SPR analyses of binding to rGPC^CD^ and rGPC^fur^ were performed using a Biacore T100 instrument (GE Healthcare) as previously described [27]. The proteins are immobilized to the biosensor chip via covalently coupled M2 anti-FLAG MAb. In most experiments, rGPC^fur^ was reconstituted into a lipid bilayer on the chip as previously described [27]. Briefly, a Biacore L1 chip bearing immobilized rGPC^fur^ in DDM-containing running buffer was rinsed with 20 mM 3-[(3 cholamidopropyl) dimethylammonio]-1-propanesulfonate (CHAPS) (Sigma) and the detergent-containing buffer was replaced by liposomes containing a 3∶1 mixture of dimyristoylphosphatidylcholine and phosphatidylcholine (DMPC:PC; Avanti Polar Lipids) in running buffer without DDM (non-detergent running buffer). By this procedure, protein-bound detergent is displaced by a lipid bilayer that forms on the hydrophobic chip [33]. At the completion of the SPR experiment, the L1 chip was regenerated with isopropanol/40 mM NaOH (2∶3 v/v) to remove lipids and associated proteins, and the M2 MAb was reconditioned using glycine:HCl, pH 3.0.

For proteolytic cleavage of rGPC^fur^, the L1 chip was incubated with 20,000 units/ml sFurin for 1 hr at 25°C in non-detergent running buffer (containing 1 µM Zn^++^). The bolus of sFurin was then washed off to achieve a stable baseline. In all binding studies, multiple concentrations of MAb (0.1–2.5 µM), sTfR (0.5–5.0 µM) and small-molecule fusion inhibitors (5–250 µM) were used to calculate the interaction parameters listed in Tables 1 and 2, respectively.

**Table 1:** Summary of rGPC^fur^ interaction parameters from SPR studies.

	k_a_ (M^−1^s^−1^)	k_d_ (s^−1^)	K_d_ (nM)^a^	rGPC^CD^ K_d_ (nM)^b^
MAb BF11	4.2×10^5^	0.007	16.66 (1.2)	10.1
MAb BF09	1.8×10^5^	0.062	344.44 (14.5)	219
MAb BE08	1.2×10^5^	0.081	675 (20.3)	125
MAb AG02	0.69×10^5^	0.085	1,231 (93.2)	583
sTfR	0.29×10^5^	0.078	2,689 (120.5)	1,296

a *K_d_* values are the means derived by fitting two or three independent biosensor datasets. The number in parenthesis is the difference between the highest and lowest *K_d_* values from individual datasets.

b *K_d_* values for rGPC^CD^ binding are taken from Thomas *et al*. [27].

**Table 2:** Summary of inhibitor-rGPC^fur^ interaction parameters from SPR studies.

Inhibitor	*k_a_* (M^−1^s^−1^)	*k_d_* (s^−1^)	*K_d_* (µM)^a^	rGPC^CD^ *K_d_* (µM)^b^
ST-294	7.9×10^3^	0.015	1.89 (0.3)	1.08
17C8	1.8×10^3^	0.031	17.22 (2.3)	9.50
ST-375	2.1×10^3^	0.02	9.52 (0.8)	3.45
ST-193	1.2×10^3^	0.02	16.66 (1.8)	10.52
ST-761	1.8×10^3^	0.02	11.11 (0.9)	8.67

a *K_d_* values are the means derived by fitting two or three independent biosensor datasets. The number in parenthesis is the difference between the highest and lowest *K_d_* values from individual datasets.

b *K_d_* values for rGPC^CD^ binding are taken from Thomas *et al*. [27].

pH-induced shedding of the G1 subunit was determined by subjecting the cleaved rGPC^fur^ to a 1 min pulse of non-detergent running buffer that had been adjusted to pH 5.0 using PIPES or sodium acetate. A new baseline was subsequently determined to assess the loss of protein from the chip. In studies using ST-294 to inhibit G1 shedding, 10–20 µM of the compound was included at all stages of the experiment. The addition of ST-294 did not affect sFurin cleavage.

### Preparation of Liposomes and Proteoliposomes

DMPC:PC liposomes used to form lipid bilayers in SPR studies were prepared as previously described [27]. For liposomal fusion assays, large unilamellar vesicles (LUVs) were prepared using a 7∶3 mixture of POPG (1-palmitoyl,2-oleoyl-sn-glycero-3-phosphoglycerol) and POPC (1-palmitoyl,2-oleoyl-sn-glycero-3-phosphocholine) (Avanti Polar Lipids). Dried lipids were hydrated in 10 mM Tris (pH 7.2), 100 mM NaCl, 50 µM ZnSO_4_ and multilamellar liposomes were formed by repeated cycles of freeze/thaw. LUVs were produced by extrusion through 100-nm pore size polycarbonate membranes (Avestin). To generate target liposomes bearing a self-quenching concentration of the fluorescent lipid rhodamine-PE (1,2-dioleoyl-sn-glycero-3-phosphoethanolamine-N-(lissamine rhodamine B sulfonyl); Avanti Polar Lipids), the lipid mix was doped with 1% of the compound. For studies of content mixing (below), liposomes included 10% cholesterol hemisuccinate and contained either 50 µM Zn^++^ (proteoliposomes) or 1 µM of the soluble zinc-sensitive fluorophore FluoZin-1 (Life Technologies) and no Zn^++^ (target liposomes). Free Zn^++^ or unincorporated FluoZin-1 was removed by dialysis or separation on a small Sepharose CL4B (Sigma) column.

Proteoliposomes bearing rGPC^fur^ were prepared by mixing rGPC^fur^ (in buffer containing 50 µM Zn^++^ and 0.05% DDM) with the appropriate LUVs at a protein:lipid molar ratio of ∼1∶25. After 30 min of incubation at room temperature, the proteoliposomes were desalted by size-exclusion chromatography using Sepharose CL4B, and extruded through a polycarbonate membrane (100-nm pore). Protein incorporation was generally >95%. Proteoliposomal rGPC^fur^ (1.5–3.0 µM protein) was cleaved prior to use in fusion assays by incubation with 0.5 µM sFurin at 20°C for 45 min in buffer containing 10 mM Tris (pH 7.2), 100 mM NaCl.

### Liposome Fusion Assays

Lipid mixing: Target liposomes containing rhodamine-PE were added to furin-treated proteoliposomes (∼1.5–3.0 µM rGPC^fur^ in 50 µM lipid) at a ratio of 1∶10, and lipid mixing was assessed intermittently by fluorescence (excitation 508 nm; emission 600 nm) using a Perkin Elmer LS55 spectrometer. pH was adjusted to 5.0 by the addition of 1 M sodium acetate, and complete dequenching of the fluorophore was determined upon solubilization in 2% Triton X-100. JUNV-specific MAbs and small-molecule fusion inhibitors were pre-incubated with proteoliposomes at a concentration of 5 and 15 µM, respectively.

Content mixing: Target liposomes containing 1 µM FluoZin-1 were added to Zn^++^-containing furin-treated proteoliposomes (∼1.5–3.0 µM protein in 50 µM lipid) at a 1∶10 ratio, and content mixing was assessed as described above (excitation 370 nm; emission 485 nm). The fluorescence signal for complete mixing was determined by solubilizing the liposomes in buffer adjusted to 50 µM Zn^++^.

### Expression of GPC in Mammalian Cells

GPC was expressed in Vero cells, with SSP and the G1G2 precursor in *trans*, using pcDNA-based plasmids and T7 polymerase provided by the recombinant vaccinia virus rTF7-3 [34]. pH-induced cell-cell fusion activity was characterized as previously described [19].

## Results

### Expression of Recombinant GPC

In previous studies, we produced and characterized the recombinant cleavage-defective JUNV GPC precursor protein [27]. rGPC^CD^ (previously referred to as icd-GPC) was purified from membranes of baculovirus-infected insect cells and found to retain its trimeric structure. We showed that the rGPC^CD^ trimer binds the JUNV TfR1 receptor [12], JUNV-neutralizing MAbs [28], and small-molecule arenavirus-specific fusion inhibitors [27]. Because the rGPC^CD^ mutant is unable to mediate membrane fusion [19], we expressed wild-type rGPC. As previously described [27], our expression strategy takes advantage of the unusual ability of SSP to associate in *trans* with the G1G2 precursor to reconstitute the native tripartite complex [18], [35]. Thus, SSP and the wild-type G1G2 precursor were co-expressed using a pFastBac-Dual dual-promoter baculovirus vector. The G1G2 open-reading frame contains a conventional signal peptide and a FLAG tag is appended at the C-terminus to facilitate purification from solubilized insect-cell membranes.

**Figure 1:**
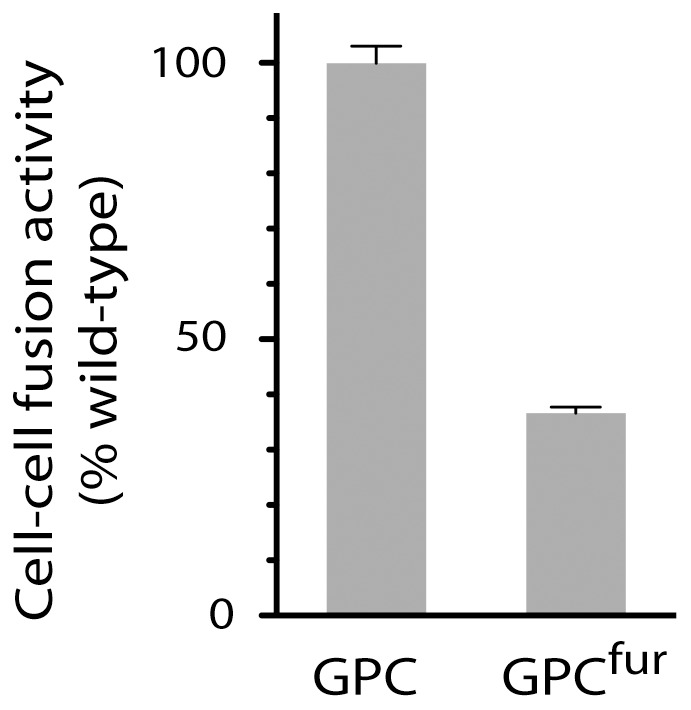
The GPC^fur^ mutant is fusogenic in mammalian cell culture. The furin cleavage-site mutation was introduced into the wild-type G1G2 precursor, which was expressed in *trans* with SSP in Vero cells. pH-induced cell-cell fusion activity relative to the wild-type GPC was determined using a vaccinia virus-based beta-galactosidase fusion-reporter assay as previously described [19]. Error bars indicate the standard error of the mean. Background signals obtained in the absence of SSP are ≤2% of wild-type.

**Figure 2:**
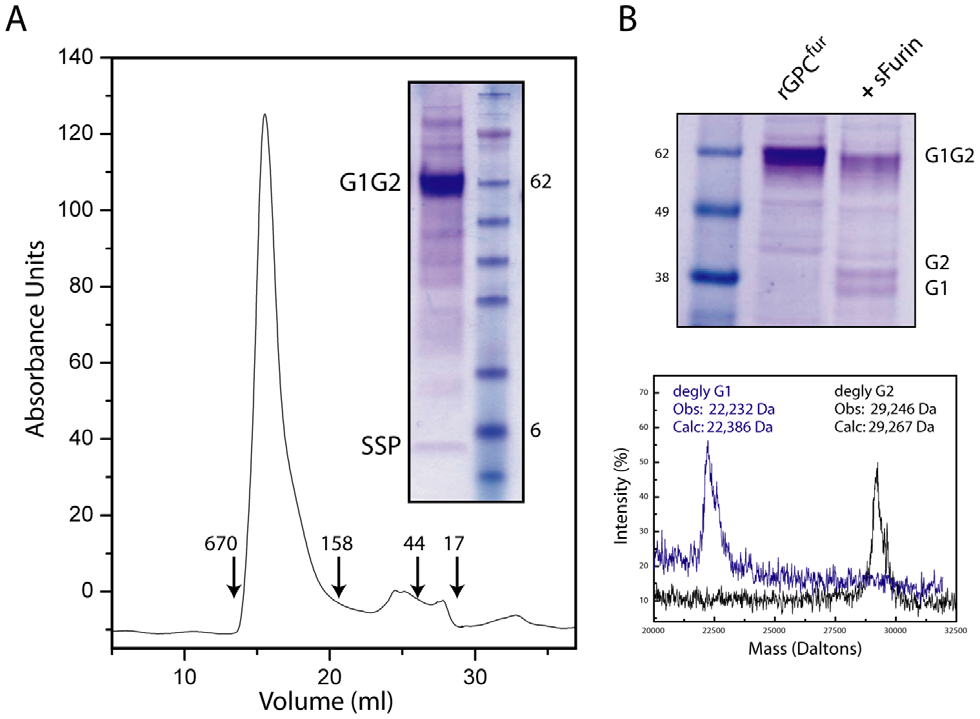
Purification and proteolytic cleavage of rGPC^fur^. **A.** Affinity-purified rGPC^fur^ was subjected to size-exclusion chromatography (SEC) and the peak fraction was analyzed by SDS-PAGE. Molecular weight markers used in SEC and SDS-PAGE are shown (in kilodaltons) and G1G2 precursor, G1 and G2 subunits and SSP are indicated. **B.** rGPC^fur^ precursor was incubated with sFurin (+sFurin) and examined by SDS-PAGE (top panel). MALDI mass spectrometry was used to determine the molecular weights of the deglycosylated (degly) G1 and G2 subunits. The calculated mass is based on the assumption that all potential glycosylation sites are used and subsequently deglycosylated.

Despite the existence of an insect S1P/SKI-1 orthologue [36], the wild-type rGPC was refractory to cleavage in insect cells. Serendipitously, several laboratories had previously reported functional GPC mutants in which the S1P/SKI-1 cleavage site (RRSLK|A) was replaced by that of furin [37], [38], a cellular protease used in the maturation of other viral envelope glycoproteins. We therefore introduced a furin recognition site (RRRKR|A) into the JUNV G1G2 precursor (GPC^fur^) and confirmed that the mutant protein was fusogenic when expressed in mammalian cells (Fig. 1). In insect cells, however, rGPC^fur^ remained refractory to cleavage (Fig. 2A), perhaps reflecting the differing specificity of the insect enzyme [39]. Based on these results, we purified the rGPC^fur^ precursor to investigate its susceptible to *in vitro* cleavage by human furin.

### Purification of the rGPC^fur^ Precursor

The rGPC^fur^ precursor was solubilized from insect-cell membranes using 1.5% DDM and purified by affinity chromatography using the C-terminal FLAG tag [27]. SDS-PAGE analysis of the purified protein confirmed SSP association in the rGPC^fur^ complex as well as the lack of proteolytic cleavage in insect cells (Fig. 2A). Similarly to rGPC^CD^ [27], the rGPC^fur^ precursor formed a relatively homogenous oligomer on size-exclusion chromatography, with an apparent molecular size consistent with a trimer (∼220 kDa) (Fig. 2A). The yield of purified rGPC^fur^ by this procedure was ∼1 mg per liter of High-Five™ cell culture.

**Figure 3:**
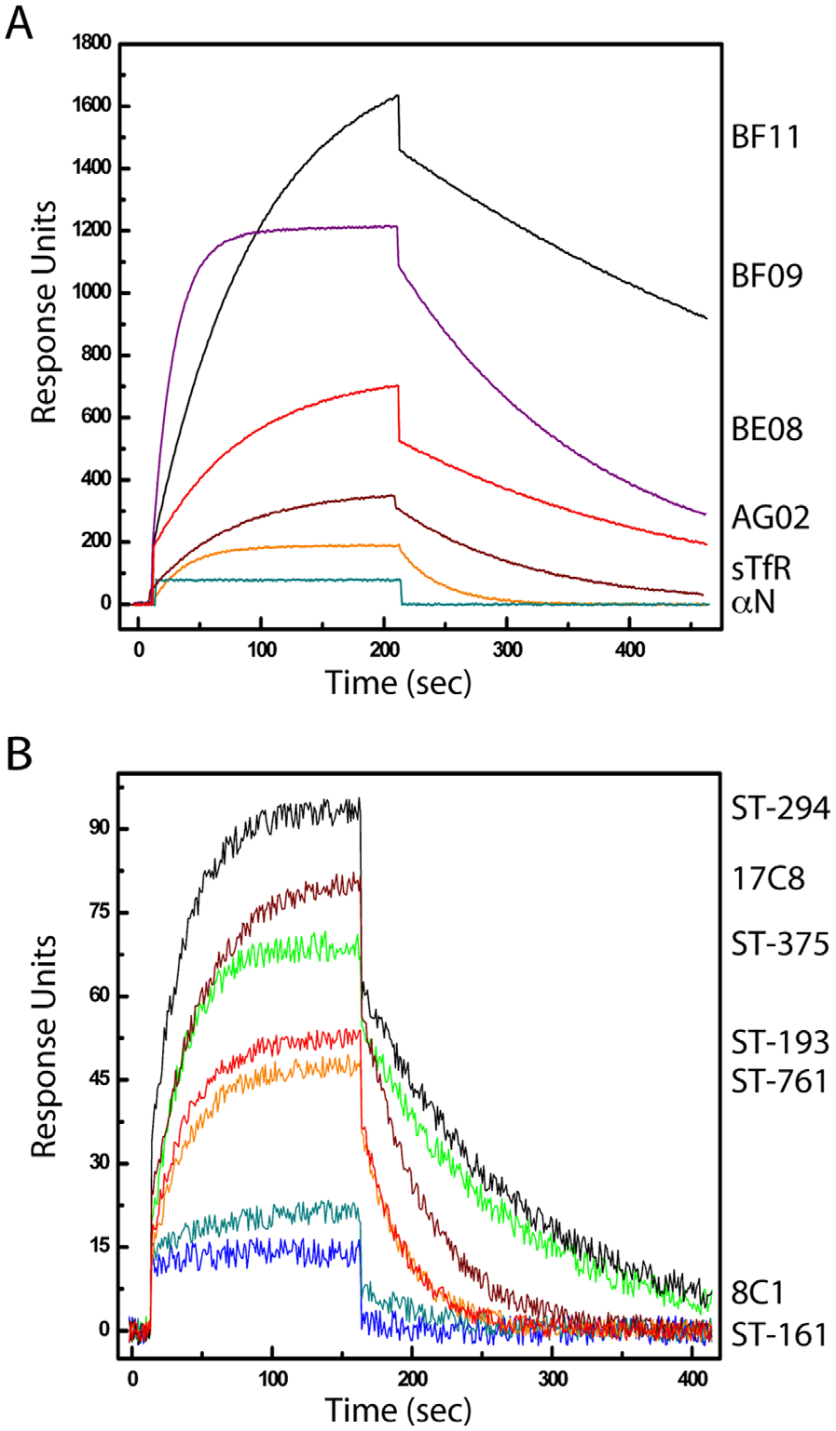
SPR studies of interactions with lipid-reconstituted rGPC^fur^. The rGPC^fur^ precursor was immobilized onto a Biacore L1 chip and reconstituted in a lipid bilayer as described in Material and Methods. Two or three concentration-dependent data sets were analyzed and sensorgram figures were generated using a five-point smoothing procedure and ORIGIN graphing software. Labels to the right are drawn to coincide with the maximum RUs achieved by the respective ligand. **A.** Binding of G1-directed MAbs (BF11, BF09, BE08, and AG02; 0.5 µM), sTfR (1.5 µM), and a nucleoprotein-directed MAb (aN; BG12). **B.** Binding of SIGA (ST-294, ST-375, ST-193, and ST-761; 150 µM) and TSRI (17C8; 100 µM) small-molecule fusion inhibitors. ST-161 and TSRI 8C1 are specific to the OW LASV and do not inhibit the NW arenavirus JUNV.

The furin-site mutation in rGPC^fur^ did not appear to affect recognition of the trimer by virus-neutralizing MAbs or the TfR1 receptor in SPR studies. Briefly, rGPC^fur^ was captured onto a hydrophobic Biacore L1 sensor chip using immobilized FLAG MAb, and subsequently reconstituted into a lipid membrane [27], [33]. Using a panel of four well-characterized virus-neutralizing MAbs directed to JUNV G1 [28], we found that the rGPC^fur^ precursor was antigenically indistinguishable from rGPC^CD^ and bound to soluble TfR1 with comparable affinity [27] (Fig. 3A and Table 1). Together, these results suggest that the purified rGPC^fur^ trimer retains important aspects of the native structure.

### Small-molecule Arenavirus-specific Fusion Inhibitors

Six chemically distinct classes of small-molecule arenavirus-specific entry inhibitors have been identified through independent high-throughput screening exercises at SIGA Technologies (SIGA) and the Scripps Research Institute (TSRI) [24]–[26], [29]. These classes differ in their specificities for NW arenaviruses (JUNV) and/or OW viruses (LASV) (see Materials and Methods) yet appear to share a common binding site on GPC [26], [27]. The compounds are thought to act through the SSP-G2 interface to stabilize prefusion GPC against activation at endosomal pH, thereby inhibiting membrane fusion [26], [27].

SPR studies were performed to determine the ability of these inhibitors to bind lipid-reconstituted rGPC^fur^. Compounds active against NW viruses (ST-294, ST-761, ST-193, and TSRI 17C8) bound to JUNV rGPC^fur^, whereas LASV-specific compounds (ST-161 and TSRI 8C1) did not (Fig. 3B). Furthermore, the dissociation constants (*K*_d_s) were similar to those determined for rGPC^CD^ (Table 2), approaching concentrations required for 50% inhibition of cell-cell fusion (IC_50_) by the wild-type GPC in mammalian cell culture [27]. Collectively, our binding studies suggest that the furin-site mutation is well tolerated, and that the rGPC^fur^ precursor produced in insect cells adopts a native-like conformation.

### Proteolytic Cleavage of Purified rGPC^fur^

For proteolytic cleavage of rGPC^fur^, we expressed recombinant soluble human furin (sFurin) in insect cells and purified the enzyme by Ni^++^-affinity chromatography [30]. sFurin digestion of the purified rGPC^fur^ precursor in nonionic detergent at 20°C resulted in only partial (∼40%) cleavage (Fig. 2B). We were unable to identify conditions to drive the digestion to completion and speculate that the engineered furin recognition site may not be optimally presented for cleavage [40]. Nonetheless, MALDI mass spectrometric analysis of the G1 and G2 subunits generated by furin digestion at 20°C confirmed cleavage at the predicted site (RRRKR|A) and the production of the wild-type G2 subunit (Fig. 2B).

### pH-induced Conformational Changes in Furin-cleaved rGPC^fur^

**Figure 4:**
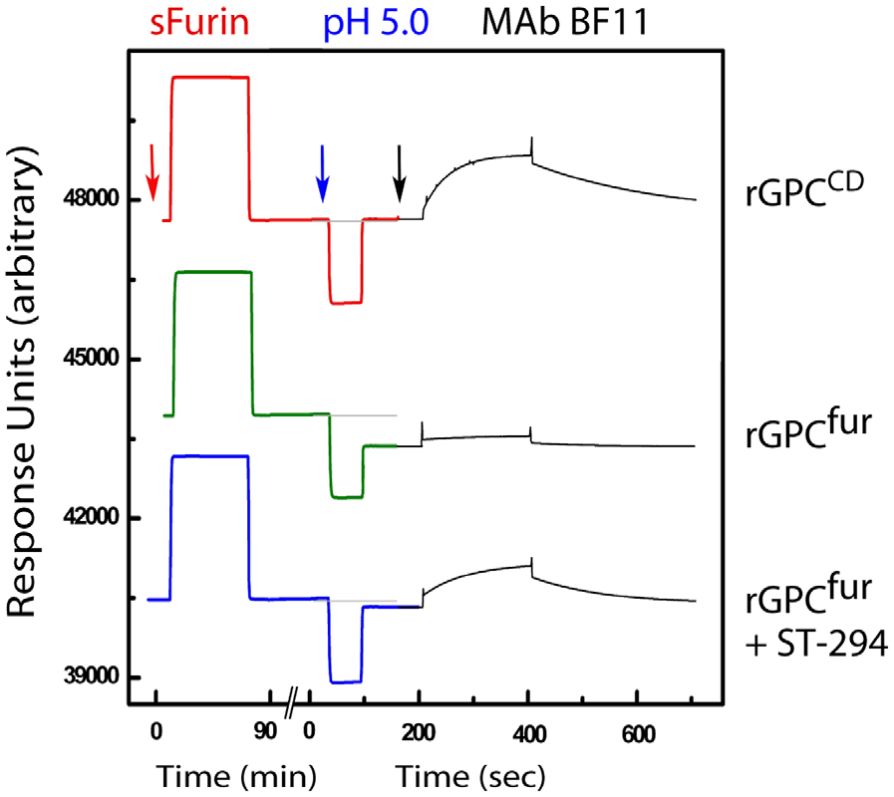
SPR analysis of pH-induced G1 shedding. rGPC was immobilized onto a Biacore L1 chip and reconstituted in a lipid bilayer as described [27]. The figure includes a composite of SPR responses from single-chip experiments in which rGPC^CD^ (top trace) or rGPC^fur^ (bottom two traces) were sequentially incubated with sFurin (red arrow), exposed to pH 5.0 (blue arrow), and probed with the G1-specific MAb BF11 (black arrow). In the lower trace, 20 µM of ST-294 was present throughout the experiment. The relative baseline before exposure to pH 5.0 is shown as a gray line and the absolute RU values on the y-axis are arbitrary.

We reasoned that cleaved rGPC^fur^ should differ from the uncleaved protein in its response to acidic pH. Fusion activation in class I fusion proteins typically results in the dissociation of noncovalently associated receptor-binding and transmembrane subunits [41]. Indeed, mammalian cells expressing GPC have been shown to shed G1 on exposure to acidic pH [26]. We therefore examined G1 shedding from cleaved rGPC^fur^ as an indication of pH-induced fusion activation. In these experiments, lipid-reconstituted rGPC^fur^ was incubated on the biosensor chip with a bolus of sFurin (Fig. 4, middle trace, left). On washing, the SPR response units (RUs) returned to the pre-digestion baseline, indicating that no protein had been lost from the chip. Following transient exposure to pH 5.0, however, the baseline signal was reduced by ∼800 RUs, consistent with pH-induced shedding of G1 (Fig. 4, middle trace, center). By contrast, identical treatment of the rGPC^CD^ precursor resulted in no loss of protein (Fig. 4, top trace). Based on the magnitude of the pH-induced loss from furin-treated rGPC^fur^, we estimate that cleavage was ∼70% complete under these conditions. Susceptibility of rGPC^fur^ to furin cleavage may be enhanced by membrane anchorage.

To confirm that the pH-induced reduction in baseline RUs reflects a loss of G1, we probed the same chip surface with a G1-specific MAb [28]. Binding of MAb BF11 to cleaved rGPC^fur^ was markedly reduced after exposure to pH 5.0 relative to the rGPC^CD^ control (Fig. 4, right), indicative of pH-induced G1 shedding. Furthermore, the loss of G1 from cleaved rGPC^fur^ was found to be inhibited by ST-294 (Fig. 4, lower trace), in agreement with results obtained using native GPC on the surface of mammalian cells [26]. We conclude rGPC^fur^ can be functionally matured *in vitro* and that the prefusion trimer is able to respond to acidic pH in a manner consistent with on-path conformational changes seen in the native GPC complex.

**Figure 5:**
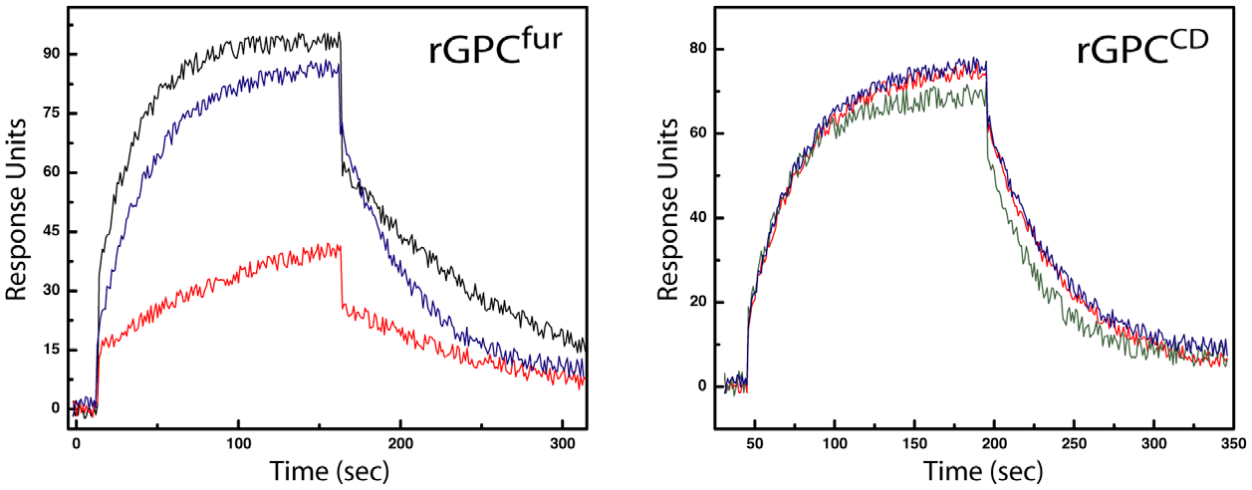
SPR studies of ST-294 binding to rGPC^fur^ following cleavage and exposure to acidic pH. A. ST-294 (100 µM) was bound to lipid-reconstituted rGPC^fur^ on a Biacore L1 chip prior to (black) and after (blue) sFurin cleavage, and following exposure of the latter to acidic pH (red). Bound ST-294 was allowed to dissociate from the complex between subsequent injections. **B.** In a similar study using immobilized cleavage-defective rGPC^CD^, ST-294 was bound prior to (blue) and following one and two sequential exposures to acidic pH (red and green, respectively).

Proteolytic cleavage of the rGPC^fur^ precursor was found to have only a minimal effect on the extent and overall affinity of ST-294 binding (*K*_d_ = 2 µM *vs.* 4 µM) (Fig. 5A). However, exposure to acidic pH markedly reduced the extent of subsequent ST-294 binding (Fig. 5A). The *K_d_* of the residual binding was unaffected and is likely attributable to uncleaved rGPC^fur^ remaining on the SPR chip. This notion is supported by observations that the cleavage-defective rGPC^CD^ can be repeatedly cycled at low pH without loss of ST-294 binding activity (Fig. 5B). These latter observations suggest that the trimeric precursor does not undergo significant irreversible change in response to acidic pH. The covalent linkage of the G1 and G2 subunits in the precursor likely constrains pH-induced conformational excursions from the initial state.

### Furin-cleaved rGPC^fur^ Mediates pH-dependent Membrane Fusion

**Figure 6:**
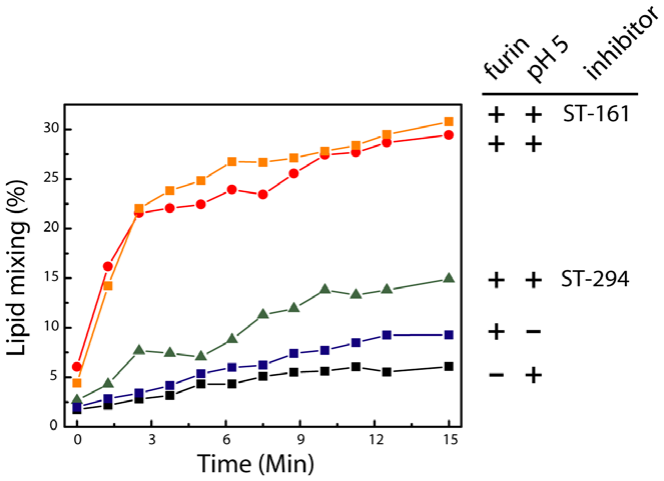
pH-induced membrane fusion by rGPC^fur^ proteoliposomes: lipid mixing. rGPC^fur^ was incorporated into POPG-POPC liposomes and mixed with target POPG-POPC liposomes doped with 1% Rhodamine-PE. In most cases, the rGPC^fur^ proteoliposomes were first treated with sFurin (as indicated by+in the first position of the labels, at right). Exposure to acidic pH at the start of the experiment (time = 0) is indicated by+in the second position of the labels. 15 µM of ST-294 or ST-161 was present prior to and during exposure to acidic pH, where indicated. Lipid mixing and the resulting dequenching of the rhodamine fluorophore were measured at 600 nm (excitation 508 nm). Complete dequenching (100%) was determined by subsequent solubilization in Triton X-100 nonionic detergent.

In order to determine whether cleaved rGPC^fur^ is able to mediate membrane fusion, we incorporated the recombinant protein into LUVs and examined the ability of the proteoliposomes to fuse with target LUVs in a pH-dependent manner. The target liposomes were doped with a self-quenching concentration of rhodamine-PE, and fusion with the proteoliposome results in dilution of the rhodamine within the merged lipids and dequenching of the fluorophore [42]. At neutral pH, the liposome mixture was stable over the course of 15 min, regardless of whether proteoliposomes had previously been incubated with sFurin (Fig. 6). Proteoliposomes bearing uncleaved rGPC^fur^ were likewise stable when exposed to acidic pH. By contrast, acidification of sFurin-cleaved rGPC^fur^ resulted in a rapid increase in fluorescence over a period of 3 min (Fig. 6). After 5 min, rhodamine-PE fluorescence approached ∼30% of the maximum obtained on complete dequenching in nonionic detergent. Importantly, pH-induced fusion was inhibited by the prior addition of ST-294, but not by the LASV-specific compound ST-161 (Fig. 6). Thus, reconstituted rGPC^fur^ is proteolytically primed and can be activated by acidic pH to mediate fusion.

**Figure 7:**
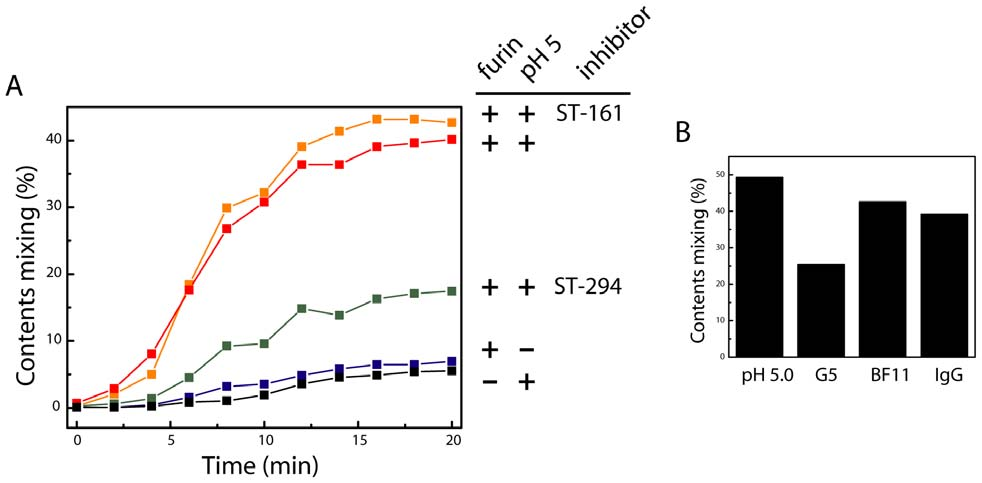
pH-induced membrane fusion by rGPC^fur^ proteoliposomes: content mixing. **A.** rGPC^fur^ was incorporated into POPG-POPC-CHS liposomes containing 50 µM Zn^++^ and these proteoliposomes were mixed with target POPG-POPC-CHS liposomes containing 1 µM FluoZin-1. The experimental treatments, labels and colors are as described in the legend of Fig. 6. Content mixing was determined by Zn^++^-induced FluoZin-1 fluorescence measured at 485 nm (excitation 370 nm). Complete mixing was determined by solubilization with Triton X-100 in buffer containing 50 µM Zn^++^. **B.** pH-induced content mixing was determined in the continued presence of MAbs F100G5, BF11 or an irrelevant IgG (5 µM, 25 µM and 25 µM, respectively). In this experiment, content mixing in the absence of MAb (pH 5.0) was ∼50%.

Rhodamine dequenching upon pH-induced activation demonstrates mixing of the liposomal lipids. At a minimum, this result indicates that cleaved rGPC^fur^ is capable of inducing hemifusion, an intermediate state in fusion where only the outer leaflets of the two membranes merge. Previous studies have shown that engineered envelope proteins linked to the cell membrane only via a lipid glycophosphatidylinositol (GPI) anchor, or in which the transmembrane domain is truncated, arrest at this hemifusion intermediate [43]–[46]. To determine whether rGPC^fur^ proteoliposomes can proceed to complete fusion, with merger of both membrane leaflets and opening of a fusion pore, we monitored pH-induced mixing of liposome contents. In these studies, target liposomes containing the Zn^++^-sensitive fluorophore FluoZin-1 were mixed with rGPC^fur^ proteoliposomes containing 50 µM Zn^++^. Complete fusion with content mixing results in an increase in FluoZin-1 fluorescence. Without sFurin-cleavage, only baseline fluorescence was detected over a 20 min period at acidic pH (Fig. 7A). Similarly, furin-treated proteoliposomes were stable at neutral pH. By contrast, acidification of the cleaved rGPC^fur^ proteoliposomes led to a rapid increase in fluorescence that was preceded by a lag period of ∼2 min (Fig. 7A). By 10 min, content mixing was ∼40% of the maximum. The lag period in content mixing may indicate a kinetic barrier for resolution of the hemifusion intermediate [47]. Our results indicate that reconstituted rGPC^fur^ mediates complete membrane fusion with content mixing. In addition, we found that rGPC^fur^-mediated fusion was markedly inhibited by ST-294, but not by the LASV-specific inhibitor ST-161 (Fig. 7A). Taken together, our studies demonstrate that recombinant rGPC^fur^ faithfully reproduces the pH-induced membrane-fusion activity of native GPC.

### Distinct Modes of Antibody-mediated Neutralization

Reconstitution of the membrane-fusion reaction provides a platform to investigate the mechanisms of antibody-mediated virus neutralization. For example, MAb F100G5 has been shown to recognize the fusion peptide of JUNV G2 [16] and, as this region is sequestered in prefusion GPC, does not bind virions and is therefore unable to inhibit entry following endocytic uptake of the particle. Liposomal fusion by rGPC^fur^ proteoliposomes was, however, inhibited when F100G5 was present in the low-pH buffer (Fig. 7B). This result recapitulates previous observations from cell-cell fusion assays [16]. By contrast, the molecular basis for virus neutralization by the G1-directed MAb BF11 [28] is unknown. Interestingly, MAb BF11 did not inhibit proteoliposomal fusion (Fig. 7B). We conclude that this MAb may block TfR binding or virion internalization. This *in vitro* model of membrane fusion offers unprecedented access for biochemical studies of GPC function and its inhibition.

## Discussion

Owing to the inherently labile nature of the prefusion envelope glycoprotein, solubilization of class I fusion proteins from the membrane typically destabilizes the trimeric structure sufficiently to cause disassembly and/or refolding to the stable postfusion state [48]–[50]. Indeed, this intrinsic instability has greatly hindered biochemical and biophysical studies on the viral fusion machinery, and frustrated efforts to design and develop native envelope glycoproteins as effective vaccine immunogens [51]. We hypothesize that the unique stability of GPC results from interaction among the nine transmembrane domains in the trimeric complex (*vs.* three in conventional class I fusion proteins), enforced by intersubunit zinc-finger structures. The fortuitous resilience of GPC offers the possibility for investigating the molecular basis of membrane fusion mediated by a membrane-anchored envelope glycoprotein *in vitro*.

Our current mechanistic understanding of viral membrane fusion was developed in part from now-classical studies of influenza virus HA (reviewed in reference [52]). High-resolution crystallographic structures of the soluble HA ectodomain in its precursor [53], prefusion [54] and postfusion [55] states serve as reference points for the current model [53], [56]. Recent studies of PIV5 F [49], [50] have reinforced central tenets of the model. These crystallographic structures, as well as those of the stable postfusion core of other class I fusion proteins, have all been determined using soluble forms of the glycoprotein ectodomain and are therefore silent with respect to the transmembrane and cytoplasmic domains. To our knowledge, this report is the first to demonstrate full reconstitution of membrane-fusion activity by a complete virus envelope glycoprotein.

Membrane anchorage is essential for membrane-fusion activity. Envelope glycoprotein ectodomains anchored in the membrane by a GPI lipid or truncated transmembrane domains are unable to drive fusion to completion, arresting at the hemifusion state [43]–[46]. Anchorage may be required to transduce forces generated upon protein refolding towards complete fusion of the two bilayers. Lateral contacts within the membrane between several envelope glycoprotein spikes may also be important for formation of the stable fusion pore. Membrane interactions of the membrane-proximal ectodomain of HIV-1 Env are involved in the fusion process and contribute to important determinants for broadly neutralizing antibodies [57], [58]. Furthermore, envelope glycoprotein membrane-spanning domains also serve as conduits to transmit information between the internal and external domains for virion assembly and virus entry [59], [60]. In GPC, amino-acid changes in the membrane-proximal and transmembrane domains have been shown to affect GPC biosynthesis and intracellular trafficking of the complex [20], [35], its sensitivity to acidic pH and small-molecule fusion inhibitors [26], [61], [62] and, indeed, viral pathogenesis [63], [64]. Further analysis of GPC structure and function using this biochemically defined *in vitro* system will advance our understanding of the conformational transitions that promote viral membrane fusion. This knowledge will directly facilitate efforts to design novel entry inhibitors for the treatment of arenavirus hemorrhagic fevers.
